# 

**Published:** 1890-01

**Authors:** 


### CONTENTS.

PAGE

Introductory, . . . . . . . . . . . . . . 1

*EDITORIALS.*

The European Epidemic, . . . . . . . . . . . 1
Scientific Medicine, . . . . . . . . . . . 2
"La Grippe," . . . . . . . . . . . . . 3
Antipyrin, . . . . . . . . . . . . . . 4
Intermittent Pulse, . . . . . . . . . . . 4
Definition of a Homœopathic Physician, . . . . . . . 5
Nothing New, . . . . . . . . . . . . . 6
Eclecticism, . . . . . . . . . . . . . 6
To Our Correspondents, . . . . . . . . . . 7
Disinfectants, . . . . . . . . . . . . . 7

*MISCELLANEOUS.*

Of the Drug Curative. Dr. P. P. Wells. Communicated by Dr. C. Carleton Smith, . . . . . . . . . . . 7
A Case Read at the Meeting of the Organon Society of Boston, December 28th. Wm. P. Wesselhœft, M. D., . . . . . 10
Magnesium-Phosphoricum and Schussler's Bio-Chemistry. D. W. Clausen, M. D., . . . . . . . . . . . . 12
Notes from Past Meetings of The Lippe Society, . . . . . 14
Our Number, . . . . . . . . . . . . . 17
The Development of the Infant. Translated by A. McNeil, M. D., 18
Are the Two Schools Alike? Edmund J. Lee, M. D., . . . . 24
What is the Simillimum? M. A. A. Wolff, M. D, . . . . . 27
Comment upon Dr. Wolff's Case. G. W. Sherbino, M. D., . . 31
Lead Poisoning. S. L., . . . . . . . . . . . 31
The Homœopathic Controversy. From *N. Y. Sun*, . . . . . 32
Bœnninghausen and Lippe. Edmund J. Lee, M. D., . . . . . 34
Argentum-Nitricum—Mental and Nervous Symptoms. W. M. Butler, M. D., . . . . . . . . . . . . . . 36
A Confession and a Warning. E. W. Berridge, M. D., . . . . 39
Homœopathy in New York City. H. Hitchcock, M. D., . . . . 45
In Memoriam—Henry Noah Martin, M. D., . . . . . . 45
Homœopathy Phrenologically Considered. G. W. Sherbino, M. D., 46
Clinical Cases. G. W. Sherbino, M. D., . . . . . . . 47

*NOTES AND NOTICES.*

Gunpowder Stains—Black Eye—He was Fond of Coffee—The Prevention of Colds—A Good Subject for Surgery, . . . . 48

